# Editorial Department

**Published:** 1911-10

**Authors:** 


### EDITORIAL DEPARTMENT.

Richard is Himself Again.—Firing all along the line this issue.

---

Alcohol; Local Option; Prohibition; the Constitution; the Declaration of Independence; the Supreme Court; the Fourth of July; Rights and Privileges; and Thomas Jefferson.—Reading Mr. Russell's "Letter to the Editor"—published herewith—has set in motion a train of thought more or less relevant to the subject treated, and while the train is still warm (it could not be otherwise, as at this writing my thermometer is 96 and my big blow-hard electric fan is dancing a jig on my desk), I will jot down some of the thoughts aforesaid.

In the first place, I am going to take issue with the immortal Thomas J., and deny that "all men are created equal." It is the biggest lie that Thomas J. ever told; and he knew it when he was writing that Fourth of July harangue; for, at that time, he was holding in bondage the black brother, and would have denied indignantly that Cuffy, Pompey, Sambo and Mose were his equals, in any respect. Had either of them said so, he would have gotten a licking. And, as to "governing with the consent of the governed,"—rats! Had Thomas J. the consent of his slaves? Did they yield their God-given, "inalienable right of life, liberty and pursuit of happiness" to Mars Tom? Imagine Sambo, approaching him, hat in hat, at the back door, and saying:

"Mars Tom, you say all men are created equal and are endowed by God with rights that can not be taken away except by consent; now, Mars Tom, how about weuns? Is we your equal?"

Mars Tom would have felt like a fool. He could only have said, "we are talking of white men."

"Well," says Sambo, "why don't you say white men in your declaration?"

Oh, glorious Columbia! the land of the free and the home of the slave! parenthetically.

Local Option.—Funny thing. Local option has come to mean "saloon or no saloon," and ye sovereign voter votes for *"local option"* or against "local option" if he desires to vote for or against the saloon. Local option means that to do or not to do a certain thing in a community is "optional" with the voters, the majority ruling. Thus, if the county commissioners wish to bond the county to build a road, a bridge, a schoolhouse, or if the city government thinks it wise to extend sewers, pave streets, built a dam, or otherwise spend the public money or bond the city, they refer it to the voters. They exercise their "option" and vote for or against the project; majority rule. Here is "initiative and referendum" in operation.

NOW, AS TO THE CONSTITUTION.—What a handy thing it is,—how convenient! Everything that is right or desirable by those who are not of your way of thinking is unconstitutional. I submit, it is unconstitutional to lock a man up or hang him. Life and liberty the Constitution guarantees to every one, and the Declaration of Independence says they are God-given and inalienable. It is unqualified.

RIGHT VS. PRIVILEGE.—Some claim that they have a right to sell liquor. If so, why do they have to pay for it? A right is inherent. It is something everyone can exercise without asking anyone's permission. A privilege is something granted by those, or some one, in authority, and, in the majority of cases, the privilege is sold,—for a money consideration. If you or anyone think that you have a right to sell liquor, try it, and see where you will land. You must purchase the privilege. And, by the bye, the government, knowing that the retailing of liquor, especially in the open (or shut) saloon, under the most vicious surroundings, and having numerous Supreme Court decisions to the effect that it is a great evil,—the greatest and most far-reaching and destructive evil of our civilization (?)—*compromises* with it, licenses it,—fosters and protects it—for money! Licenses the agency which, more than any other, operates to break up homes, to make divorces, to make widows and orphans, lunatics, criminals, paupers, prostitutes, epileptics, imbeciles and consumptives, and that, too, in the face of the demonstrable fact that the cost of the care of the victims of alcohol exceeds by about three hundred per cent the entire revenue from liquor licenses.

MR. RUSSELL quotes some Supreme Court opinions which, in my opinion, demonstrate the absolute idiocy of continuing a policy so destructive of everything that is desirable in any community, and which strikes at the very roots of our civilization,—race integrity!

